# Loss of Metal Ions, Disulfide Reduction and Mutations Related to Familial ALS Promote Formation of Amyloid-Like Aggregates from Superoxide Dismutase

**DOI:** 10.1371/journal.pone.0005004

**Published:** 2009-03-27

**Authors:** Zeynep A. Oztug Durer, Jeffrey A. Cohlberg, Phong Dinh, Shelby Padua, Krista Ehrenclou, Sean Downes, James K. Tan, Yoko Nakano, Christopher J. Bowman, Jessica L. Hoskins, Chuhee Kwon, Andrew Z. Mason, Jorge A. Rodriguez, Peter A. Doucette, Bryan F. Shaw, Joan Selverstone Valentine

**Affiliations:** 1 Department of Chemistry and Biochemistry, California State University Long Beach, Long Beach, California, United States of America; 2 Department of Physics and Astronomy, California State University Long Beach, Long Beach, California, United States of America; 3 Department of Biological Sciences, California State University Long Beach, Long Beach, California, United States of America; 4 Department of Chemistry and Biochemistry, University of California Los Angeles, Los Angeles, California, United States of America; Mental Health Research Institute of Victoria, Australia

## Abstract

Mutations in the gene encoding Cu-Zn superoxide dismutase (SOD1) are one of the causes of familial amyotrophic lateral sclerosis (FALS). Fibrillar inclusions containing SOD1 and SOD1 inclusions that bind the amyloid-specific dye thioflavin S have been found in neurons of transgenic mice expressing mutant SOD1. Therefore, the formation of amyloid fibrils from human SOD1 was investigated. When agitated at acidic pH in the presence of low concentrations of guanidine or acetonitrile, metalated SOD1 formed fibrillar material which bound both thioflavin T and Congo red and had circular dichroism and infrared spectra characteristic of amyloid. While metalated SOD1 did not form amyloid-like aggregates at neutral pH, either removing metals from SOD1 with its intramolecular disulfide bond intact or reducing the intramolecular disulfide bond of metalated SOD1 was sufficient to promote formation of these aggregates. SOD1 formed amyloid-like aggregates both with and without intermolecular disulfide bonds, depending on the incubation conditions, and a mutant SOD1 lacking free sulfhydryl groups (AS-SOD1) formed amyloid-like aggregates at neutral pH under reducing conditions. ALS mutations enhanced the ability of disulfide-reduced SOD1 to form amyloid-like aggregates, and apo-AS-SOD1 formed amyloid-like aggregates at pH 7 only when an ALS mutation was also present. These results indicate that some mutations related to ALS promote formation of amyloid-like aggregates by facilitating the loss of metals and/or by making the intramolecular disulfide bond more susceptible to reduction, thus allowing the conversion of SOD1 to a form that aggregates to form resembling amyloid. Furthermore, the occurrence of amyloid-like aggregates *per se* does not depend on forming intermolecular disulfide bonds, and multiple forms of such aggregates can be produced from SOD1.

## Introduction

More than sixty human diseases are accompanied by the formation of protein aggregates called amyloid [1]. These include a number of neurodegenerative diseases, such as Alzheimer's disease, Parkinson's disease, Huntington's disease, and Creutzfeldt-Jakob (prion) disease. In each amyloid disease, a normally soluble protein forms insoluble fibrillar structures that bind the dyes thioflavin T (ThT), thioflavin S, and Congo Red, and, in many cases, display an X-ray diffraction pattern suggesting a “cross-beta structure”, in which the β-strands are oriented perpendicular to the long axis of the fiber. The amyloid deposits associated with a particular disease may be either extracellular or intracellular.

Amyloid deposits may be involved in the neurodegenerative disease amyotrophic lateral sclerosis (ALS), commonly known as Lou Gehrig's disease. Approximately 2% of ALS cases are caused by mutations in the gene encoding the anti-oxidant enzyme copper-zinc superoxide dismutase (SOD1). These mutations represent one of the few known causes of ALS and underlie the most well-studied mouse models of this devastating disease. Clearly, much can be learned about the molecular underpinnings of pathology in ALS by studying the SOD1-linked forms of the disease. A growing body of evidence supports the hypothesis that many, if not all, of the SOD1 mutations act by increasing the tendency of SOD1 to aggregate (reviewed in [2]–[5]), and some findings suggest the involvement of amyloid in pathology. Electron microscopy has revealed a fibrillar morphology of the SOD1 aggregates found in motor neurons of FALS patients [6], in COS cells expressing mutant but not wild-type (WT) SOD1 [7], in neuroblastoma cells expressing ALS mutant SOD1 which were subjected to endoplasmic reticulum stress [8], and in transgenic mice expressing ALS mutant SOD1 [6], [9], [10]. Furthermore, transgenic mice expressing mutant SOD1 proteins have neuronal inclusions which bind the amyloid-specific dye thioflavin S [11]–[13].

SOD1 is a homodimer, each polypeptide chain containing 153 amino acids with one bound Cu^2+^ and one bound Zn^2+^ ion. Each chain folds into an eight-stranded beta barrel that is flanked on one side by a number of loops which contain the metal binding sites. There are four cysteines, C6 and C111 present as free sulfhydryls, and C57 and C146 joined by a disulfide bond which links one of the loops to the beta barrel. In the absence of coordinated copper and zinc, the beta barrel and dimer interface remain intact, but the metal binding loops are disordered [5]. When neither metals nor the intramolecular disulfide bond is present, SOD1 exists as a monomer [14]–[15]. More than 100 mutations in the SOD1 gene, scattered throughout the polypeptide chain, have been linked to FALS [2]–[3]. The ALS-linked SOD1 mutants have been grouped into two general classes, “wild-type-like mutants” resulting from mutations in the beta barrel or dimer interface, which generally retain high levels of catalytic activity, and metal-binding region mutants, resulting mostly from mutations in the loops, which generally have much less catalytic activity and are isolated with lower metal content than WT SOD1 [3].

In the present study we demonstrate that under appropriate conditions a variety of biophysically diverse SOD1 species all form insoluble aggregates which are identified as amyloid fibrils by a number of different criteria. While the metalated WT enzyme does not aggregate at neutral pH, either removal of copper and zinc or reduction of the intramolecular disulfide bond is sufficient to trigger aggregation. Previous publications have shown that some forms of SOD1 generate fibrillar aggregates that bind ThT upon extended incubation at acidic pH [16], and that both WT and mutant apo-SOD1 can form non-fibrillar disulfide-bonded oligomers that bind ThT [17]–[18]. This study demonstrates further that SOD1 can form fibrillar aggregates with spectroscopic and dye-binding properties characteristic of amyloid *in vitro* at physiological pH, ionic srength and temperature. In our results, intermolecular disulfide bonds are not required for forming amyloid-like aggregates from SOD1, since such aggregates can also be produced from SOD1 mutants lacking free cysteines, and since, under defined conditions, SOD1 variants that do contain free cysteines can be shown to form amyloid-like aggregates lacking intermolecular disulfide bonds. A number of mutations related to FALS appear to promote amyloid formation by facilitating the loss of metals and/or by making the intramolecular disulfide bond more susceptible to reduction.

## Results

### Metal Content of Protein Preparations

The metal contents of the purified SOD1 proteins that were metalated *in vivo* and used in this study as they were isolated (“as isolated” proteins) are presented in Table 1. In addition to the wild-type protein, they include proteins with mutations related to ALS and “AS” proteins lacking free cysteines. The AS mutant, C6A/C111S, is a “pseudo-WT” SOD1 in which both free cysteines have been removed by mutation, with the buried cys6 mutated to alanine and the surface cys111 changed to serine. AS/A4V, AS/G93A and AS/G85R have ALS-related mutations in the same AS background. These AS proteins have been used by many investigators studying SOD1; AS-SOD1 has a stability similar to that of WT SOD1 but melts reversibly, while WT-SOD1 melts irreversibly, presumably because of disulfide-induced aggregation following thermal unfolding [19]–[21]. None of the proteins analyzed in this study had a full complement of copper and zinc, consistent with previous reports using similarly expressed and purified SOD1 [22]). The zinc contents of most of the proteins were close to or greater than two per dimer; SOD1 is frequently isolated with zinc binding partially to the copper sites of the enzyme [23]–[24] in addition to the normal zinc binding sites. The copper content was lower, ranging from 0.07 to 0.74 per dimer (Table 1).

**Table 1:** Metal contents of SOD1 preparations.

SOD1	Location	Cu	Zn	Sum
WT		0.48	3.06	3.54
AS (C6A/C111S)		0.46	3.06	3.52
D101N	beta barrel	0.62	2.36	2.98
E100G	beta barrel	0.54	2.40	2.94
G93A	beta barrel	0.62	2.24	2.86
AS/G93A	beta barrel	1.00	1.48	2.48
L38V	beta barrel	0.72	1.48	2.20
C146R	beta barrel (disulfide)	0.74	2.58	3.32
A4V	dimer interface	0.40	2.40	2.80
AS/A4V	dimer interface	0.36	2.12	2.48
I149T	dimer interface	0.22	1.44	1.66
D125H	metal-binding region	0.16	1.86	2.02
G85R	metal-binding region	0.24	2.96	3.20
AS/G85R	metal-binding region	0.39	1.56	1.95
H46R	metal-binding region	0.20	1.26	1.46
H80R	metal-binding region	0.10	0.40	0.50
S134N	metal-binding region	0.07	1.62	1.69

Analysis was by ICP-MS. Results are presented as metal atoms per dimer.

As stated in the Introduction, the beta barrel and dimer interface mutants belong to the class of “wild-type-like” mutants, which have metal content and catalytic activity close to that of WT SOD1, while “metal-binding-region” mutants have significantly reduced metal content and catalytic activity. In agreement with previous results, all the beta barrel mutants except L38V had a total of 2.8 to 3.0 metals per dimer, close to the value of 3.54 for WT. Of the metal-binding-region mutants D125H, H46R and H80R had greatly reduced metal contents (with only 25% of the metal sites occupied in H80R), while S134N and G85R had metal contents close to that of WT. Of the two dimer interface mutants, A4V was similar in metal content to other wild-type-like mutants, while I149T had a lower metal content. Three of the mutants lacking free sulfhydryl groups, AS, AS/A4V and AS/G93A, had total metal contents similar to the corresponding proteins with a normal cysteine content, while the metal content of AS/G85R was lower than that of G85R.

We prepared metal-free apo-proteins by extended dialysis against EDTA. ICP-MS analysis on individual preparations confirmed that extensive dialysis against EDTA effectively and reproducibly removed all the Cu and Zn from the enzyme.

In order to obtain a set of ALS mutant and WT SOD1 proteins with uniform contents of Cu and Zn, we attempted to remetalate the apo-SOD1 preparations by a published method that has been frequently used successfully with SOD1 expressed in bacteria [25]. We found, however, that this procedure failed to produce either fully metalated or fully active SOD1 proteins. We found alternative remetalation procedures that led successfully to fully metalated and fully active proteins, but these preparations failed to form any aggregates under conditions that promoted aggregate formation from the as-isolated preparations (data not shown). The failure to aggregate could be attributed to irreversible oxidation of one of the free cysteine residues. (See Text S1 and Figure S1 for details.) We therefore concluded that the use of remetalated proteins for *in vitro* aggregation studies was not feasible. For most experiments in this study, we instead used the “as-isolated” proteins which had been metalated in yeast prior to isolation. These preparations will be referred to as “metalated” SOD1 in the discussion that follows.

### Metalated WT SOD1 Forms Amyloid at pH 3

**Figure 1:**
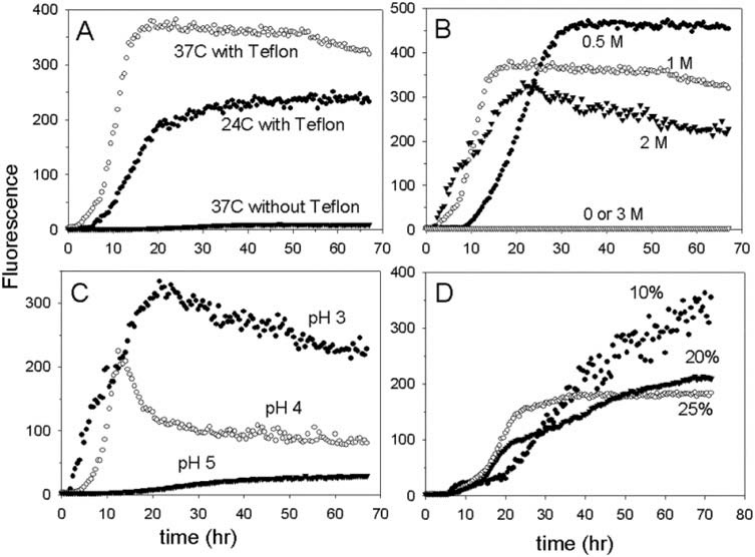
Effect of incubation conditions on amyloid formation. SOD1 at a concentration of 1 mg/ml was incubated with agitation in the Fluoroskan microplate reader for measurement of ThT fluorescence. A) Effect of temperature and agitation with a Teflon ball. Incubation buffer was 50 mM citrate, 0.1 M NaCl, 2 M guanidine, pH 3. Open circles, 37°C with Teflon balls; solid circles, 24°C with Teflon balls; open triangles, 37°C without Teflon balls. B) Effect of guanidine. Incubation buffer was 50 mM sodium citrate, 0.1 M NaCl, pH 3, containing 0 (solid triangles), 0.5 M (solid circles), 1 M (solid squares), 2 M (open inverted triangles), or 3 M (open diamonds) guanidine hydrochloride. C) Effect of pH. Incubation buffer was either 50 mM sodium citrate, pH 3 (solid circles), 50 mM sodium formate, pH 4 (open circles), or 50 mM sodium acetate, pH 5 (solid triangles), plus 0.1 M NaCl and 2 M guanidine hydrochloride. D) Effect of acetonitrile. Incubation buffer was 50 mM sodium citrate, 0.1 N NaCl, pH 3, containing 10% (solid circles), 20% (solid triangles), or 25% (open circles) acetonitrile. No amyloid was formed at 30% acetonitrile (not shown).

SOD1 solutions were incubated in the presence of 10 µM ThT, a dye that binds specifically to amyloid structures [26]–[27]. Incubations were performed in a microplate reader, as described in “Materials and Methods”, and formation of amyloid was monitored by the increase in ThT fluorescence. When WT SOD1 at a concentration of 1 mg/ml (32 µM dimer or 65 ) was incubated at either 24°C or 37°C in 50 mM sodium citrate, 0.1 M NaCl, 1 M guanidine hydrochloride, pH 3, the fluorescence remained close to zero for a lag time of a few hours, then increased until a plateau was reached. (Figure 1A). The fluorescence values at the plateau were similar to those observed in incubations of α-synuclein at the same protein concentration under conditions which have been shown to promote formation of amyloid [28]. Similar results were obtained when the buffer was formate instead of citrate, a finding which rules out the possibility that amyloid formation at pH 3 requires the presence of a buffer anion capable of coordinating copper or zinc. The amyloid character of the incubation product was confirmed by a variety of morphological and spectroscopic criteria (see below). In the remainder of this article, we use the term “amyloid” to refer to these aggregates. In incubations for as long as one week, amyloid formation was observed only when the solutions were agitated with a Teflon ball. The plateau fluorescence was higher and the lag time shorter at 37°C than at 24°C. It should be noted that amyloid formation from SOD1 at acidic pH (3.5) was also reported by DiDonato et al. [16].

At both temperatures amyloid was formed at guanidine concentrations ranging from 0.5 to 2 M, but not at 3 M (data for 37°C is shown in Figure 1B). Since the lag time was longer at 0.5 M guanidine, a concentration of 1 M and a temperature of 37°C were used in most experiments. No amyloid formed when NaCl was substituted for guanidine hydrochloride (data not shown), indicating that the effect of guanidine hydrochloride is related to its chaotropic properties and not simply to its effect on the ionic strength.

Amyloid formed within a restricted pH range. Much less amyloid formed when the pH was increased from 3 to 4, and only a very small amount of amyloid formed at pH 5 with a much longer lag time (Figure 1C). No amyloid formed at pH 2 or at pH 6 and above.

Acetonitrile could substitute for guanidine hydrochloride in promoting amyloid formation. Concentrations of 10–30% acetonitrile (but not higher concentrations) were effective (Figure 1D). Trifluoroethanol at concentrations from 10 to 30% did not promote amyloid formation (data not shown).

### Removal of Copper and Zinc Allows Amyloid Formation at Neutral pH

Because acidic pH causes the dissociation of metals from SOD1, we reasoned that the ability of WT SOD1 to form amyloid at low pH could be due, at least in part, to the loss of metal ions. We hypothesized that metal-free SOD1 might therefore be able to form amyloid at higher values of pH that are more physiologically relevant. The kinetic parameters for amyloid formation from metalated and apo-SOD1 at various pH values in the presence of 1 M guanidine are summarized in Table 2. As shown above (Figure 1C), the yield of amyloid from metalated WT-SOD1 was sharply dependent on pH, with considerably less amyloid at pH 4 or 5 compared to pH 3 and no amyloid formed at pH 6 or 7. In contrast, there was little variation in either the amplitude or the lag time for apo-SOD1 as the pH was varied between 3 and 7. This suggests that acidic pH is needed for amyloid formation mainly because it promotes the loss of metals. Most strikingly, apo-SOD1 formed amyloid at pH 6 and 7, while no conditions have been found which allow amyloid formation from metalated SOD1 with its intramolecular disulfide bond intact at pH greater than 5.

**Table 2:** Kinetic parameters of amyloid formation from metalated and apo-SOD1 in 1 M guanidine.

pH	Metalated SOD		Apo-SOD	
	amplitude	lag (hr)	amplitude	lag (hr)
3	352±180	9±2	273±93	7±4
4	62±23	3±1	162±74	19±11
5	26±10	69±38	140±51	16±4
6	0	-----	177±48	62±10
7	0	-----	248±109	26±8

SOD1 was incubated in buffers containing 50 mM citrate pH 3, formate pH 4, acetate pH 5, succinate pH 6, or MOPS pH 7, plus 0.1 M NaCl and 1 M guanidine. The time course of ThT fluorescence was fit to a sigmoidal equation and the amplitude and lag time determined as described in “Materials and Methods”. Dashes indicate that no amyloid was formed.

**Figure 2:**
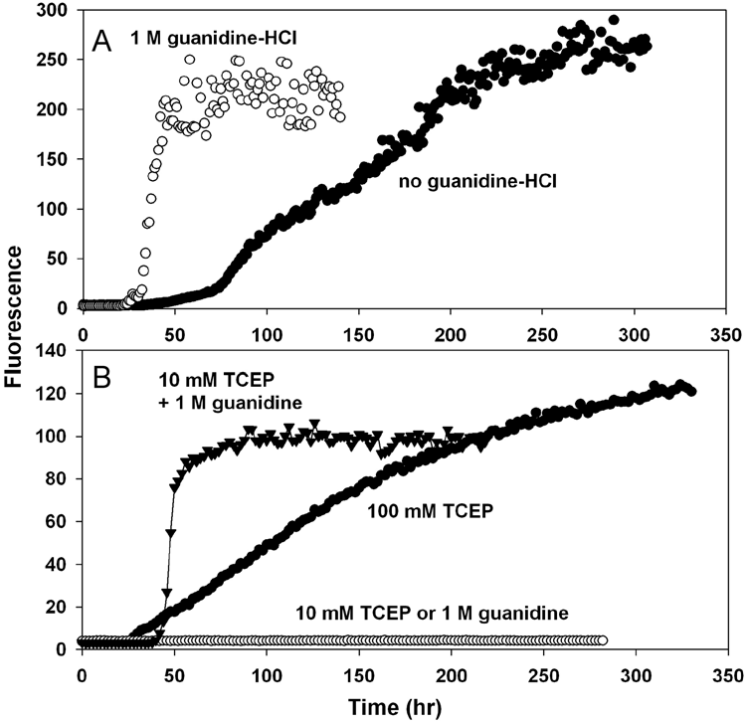
Amyloid formation from apo- and reduced WT SOD1 at pH 7. 2A) Apo-SOD1 was incubated in 50 mM MOPS, 0.1 M NaCl, 1 mM EDTA, pH 7, containing zero (solid circles) or 1 M (open circles) guanidine. 2B) Reduced SOD1. The incubation mixtures contained 50 mM MOPS, 0.1 M NaCl with 10 mM TCEP (open circles) or 1 M guanidine hydrochloride (open circles) or 100 mM TCEP (solid circles) or 10 mM TCEP/1 M GuanHCl (solid inverted triangles).

Apo-SOD1 also formed amyloid at pH 7 in the absence of guanidine. The lag times were longer than those observed for apo-SOD1 with 1 M guanidine, and the increase in fluorescence was slower and more gradual (Figure 2A). For most samples, the ThT fluorescence was still increasing after 300 hours. Addition of acetonitrile had no effect on the time course of amyloid formation from apo-SOD1 at pH 7 (data not shown).

**Figure 3:**
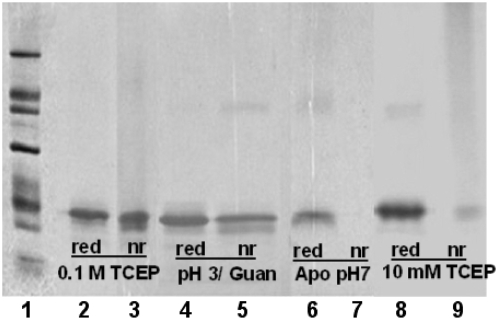
Detection of intermolecular disulfide bonds in SOD1 amyloid prepared under different conditions. Amyloid produced under each set of conditions was isolated by pelleting and subjected to SDS-PAGE in sample buffers containing 2% mercaptoethanol (“red”) or lacking any reducing agent (“nr”). Lane 1 is MW standards: 66 K, 45 K, 36 K, 29 K, 24 K, 20 K, and 14.2 K. Lanes 2 and 3, metalated SOD1 in 50 mM MOPS, 0.1 M NaCl, 0.1 M TCEP, pH 7; lanes 4 and 5, metalated SOD1 in 50 mM sodium citrate, 0.1 M NaCl, 1 M guanidine hydrochloride, pH 3; lanes 6 and 7, apo-SOD1 in 50 mM MOPS, 0.1 M NaCl, pH 7; lanes 8 and 9, metalated SOD1 in 50 mM MOPS, 0,1 M NaCl, 1 M guanidine hydrochloride, 10 mM TCEP, pH 7.

Amyloid formed from apo-SOD1 at pH 7 was stabilized against disassembly by intermolecular disulfide bonds. When the aggregated apo-WT-SOD1 was recovered by pelleting and examined by sodium dodecyl sulfate-polyacrylamide gel electrophoresis (SDS-PAGE), the protein entered the gel only when mercaptoethanol was present in the sample buffer (Figure 3, lanes 6 and 7). This is in contrast to amyloid formed at pH 3 and 1 M guanidine, in which the monomer band was only slightly less intense under nonreducing conditions than under reducing conditions, demonstrating that little if any intermolecular disulfides had formed at pH 3 (Figure 3, lanes 4 and 5). This pH dependence is in accord with the well-characterized pH dependence of disulfide formation [29].

### Reduction of the Intramolecular Disulfide Bond Promotes Amyloid Formation from Metalated SOD1

The intramolecular disulfide bond of SOD1 can be cleaved by extended incubation with high concentrations of reducing agents [30], converting SOD1^S-S^ to SOD1^2SH^. When SOD1 was incubated with 10 mM tris(2-carboxyethyl)phosphine (TCEP) and 1 M guanidine at neutral pH, there was a lag of about 45 hours followed by a rise in ThT fluorescence (Figure 2B); no amyloid was formed at pH 7 with 10 mM TCEP or 1 M guanidine alone. The use of 0.1 M TCEP allowed slow formation of amyloid at pH 7 in the absence of guanidine.

The presence of disulfide bonds in the aggregated material was dependent on the TCEP concentration during the incubation. Amyloid prepared in 1 M guanidine and 10 mM TCEP showed very little material entering an SDS gel unless mercaptoethanol was present (Figure 3, lanes 8 and 9). On the other hand, when the TCEP concentration was 0.1 M, protein from the aggregates readily entered the gel (Figure 3, lanes 2 and 3), indicating that SOD1 can form amyloid lacking disulfide bonds at neutral pH. Apparently, at the lower concentration TCEP initially cleaves the intramolecular disulfide bond but then is consumed as a result of reaction with dissolved oxygen, after which intermolecular disulfide bonds are allowed to form, while at the higher TCEP concentration the persistence of reduced TCEP throughout the incubation insures that only amyloid lacking intermolecular disulfide bonds is produced. The ability of SOD1 to form amyloid lacking intermolecular disulfide bonds is also suggested by the fact that AS-SOD1, which lacks two of the four cysteines, forms amyloid in greater yield than WT SOD1 upon incubation with 0.1 M TCEP (see “Effect of Mutations on Fibrillation of Metalated SOD1 in 0.1 M TCEP at pH 7” below).

### Electron Microscopy and Atomic Force Microscopy of Amyloid

**Figure 4:**
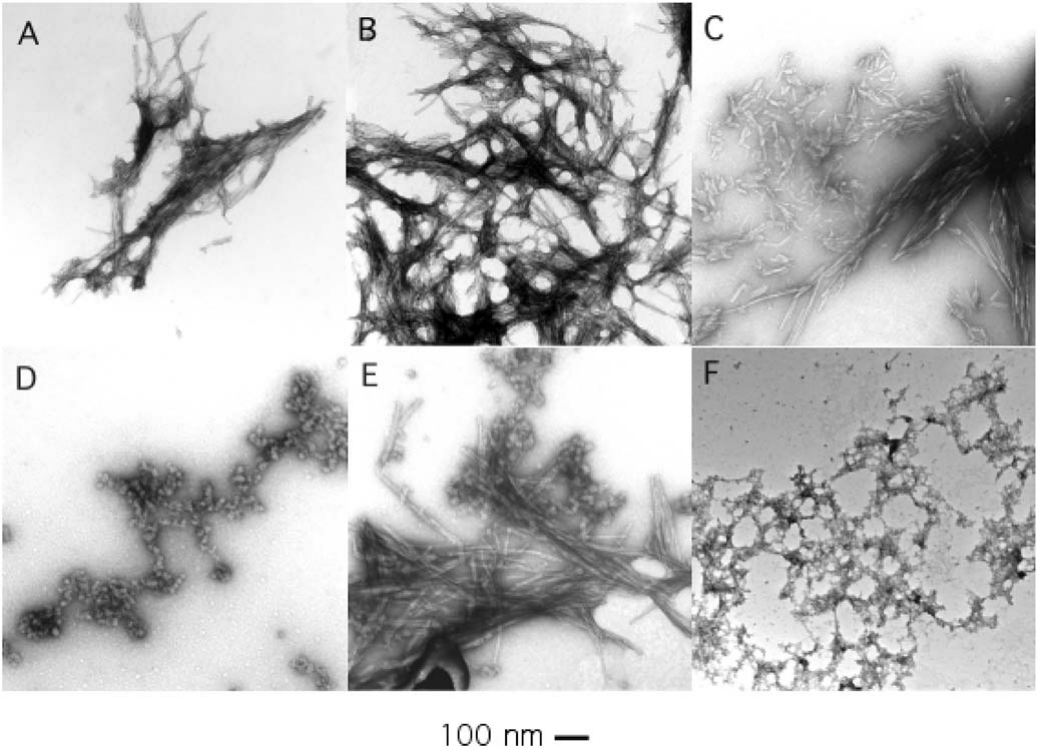
Electron microscopy of SOD1 amyloid formed at pH 3. SOD1 was incubated in 1 M guanidine hydrochloride, pH 3 at 37°C (A–B) or 24°C (C–F). Samples are WT SOD1 (A,B,C,F) or G93V (D,E).

The amyloid character of the ThT-positive aggregates was confirmed by electron microscopy. After incubation in 1 M guanidine at pH 3 and 37°C, nearly all of the protein was present as fibrils with diameters of 5 to 10 nm (some as large as 14 nm) and lengths typically 0.5 to 3 µm (Figure 4A). Often the fibrils aggregated with each other to form a meshlike network (Figure 4B). In some samples short fibrils having a distinct substructure were observed (Figure 4C). The morphology of fibrils prepared from WT SOD1 and from ALS mutant SOD1 proteins were similar. Atomic force microscopy of these preparations revealed fibrillar structures with diameters ranging from 4–14 nm (average 7.9±2.6) and lengths ranging from 0.5–3 µm (Figure S2). There appeared to be a bimodal distribution of diameters, with fibrils of 5 nm or 10 nm in diameter being most common.

Under conditions which were less than optimal for amyloid formation, as measured by the plateau value of the ThT fluorescence, nonfibrillar material was also observed in electron micrographs. For example, when the incubation was performed at 24°C at pH 3, we frequently observed spherical structures with an average diameter of about 13 nm (Figure 4D), often together with fibrils (Figure 4E) or weblike networks of thinner, less well-defined fibrillar material (Figure 4F).

Amyloid fibrils were also observed in aggregates derived from apo-SOD1 and disulfide-reduced SOD1. Long fibrils, short fibrils and meshlike networks were observed under different incubation conditions (Figure S3). Spherical structures were present in amyloid prepared from apo-SOD1 but not in amyloid prepared from TCEP-treated SOD1.

### Spectroscopic Characterization of Amyloid

The amyloid character of the aggregated material was verified by a number of spectroscopic methods. Binding of the amyloid-specific dye Congo red was demonstrated both by a shift in the absorbance maximum of the dye [31]–[32] (Figure S4A) and by birefringence as monitored by polarization microscopy [33]–[34] (Figure S4B). In addition, circular dichroism (CD) (Figure S4C) and infrared (IR) (Figure S4D) spectra characteristic of amyloid were observed. The trough in the circular dichroism spectrum shifted from 209 nm for soluble SOD1 to 220–222 nm for SOD1 amyloid. A similar spectrum has been observed for amyloid formed from a number of other proteins [35]–[40]. The infrared amide I peak shifted from 1644 cm^−1^ to 1631 cm^−1^, indicating a significant reorganization of the beta sheet characteristic of amyloid [41]. Similar spectra were obtained for amyloid prepared from both metalated and apo-SOD1 under a variety of incubation conditions. This suggests that amyloid produced under different sets of conditions have a common structural organization. Fluorescence data indicate that tryptophan 32 becomes buried upon amyloid formation and that SOD1 amyloid does not have significant amounts of solvent-exposed hydrophobic clusters (see Text S2 for details).

### ALS Mutations Enhance Amyloid Formation Under Some but Not All Incubation Conditions

In order to examine the effect of ALS mutations on the tendency of SOD1 to form amyloid, we compared the properties of wild-type and mutant proteins under each of the three sets of conditions found to promote amyloid formation from WT SOD1.

### Effect of Mutations on Fibrillation of Metalated SOD1 at Acidic pH

ALS mutations did not stimulate amyloid formation from metalated SOD1 in 1 M guanidine at pH 3. On the contrary, the yield of amyloid was as great for WT SOD1 as for any of the mutants tested, and the lag time for WT SOD1 was among the shortest of any of the proteins examined (see Table 3).

**Table 3:** Kinetic Parameters for Amyloid Formation from Metalated SOD1 at Acidic pH

	pH 3		pH 5				Location	Cu	Zn	Sum
[guanidine]	1 M		1 M		2 M					
SOD1	amplitude	lag (hr)	amplitude	lag (hr)	amplitude	lag (hr)				
WT	352±180	9±2	26±10	69±38	22±9	17±4		0.48	3.06	3.54
D101N	182±59	29±3	9±5	24±8	39±16	55±7	BB	0.62	2.36	2.98
E100G	337±226	26±14	15.3±0.3	85±29	90±47	31±10	BB	0.54	2.40	2.94
G93A	141±59	33±14	8±4	1±2	81±18	11±1	BB	0.62	2.24	2.86
L38V	350±105	15±6	----	----	85±54	16±4	BB	0.72	1.48	2.20
A4V	126±54	24±7	22±5	86±30	39±22	19±5	DI	0.40	2.40	2.80
I149T	132±39	33±17	100±33	47±13	37±10	31±16	DI	0.22	1.44	1.66
D125H	85±68	34±15	88±105	42±22	----	----	MBR	0.16	1.86	2.02
G85R	278±82	12±3	68±16	40±27	57±14	26±7	MBR	0.24	2.96	3.20
H46R	264±228	29±13	124±84	7±3	41±24	45±19	MBR	0.20	1.26	1.46
H80R	190±41	9±5	209±53	38±9	66±18	14±4	MBR	0.10	0.40	0.50
S134N	64±19	6±1	----	----	----	----	MBR	0.07	1.62	1.69

SOD1 was incubated at pH 3 or pH 5 in 1 or 2 M guanidine, as indicated. The time course of ThT fluorescence was fit to a sigmoidal equation and the amplitude and lag time determined as described in “Materials and Methods”. Dashes indicate that no amyloid was formed. Location: BB = beta barrel; DI = dimer interface; MBR = metal-binding region. Cu and Zn contents are metal atoms per dimer.

**Figure 5:**
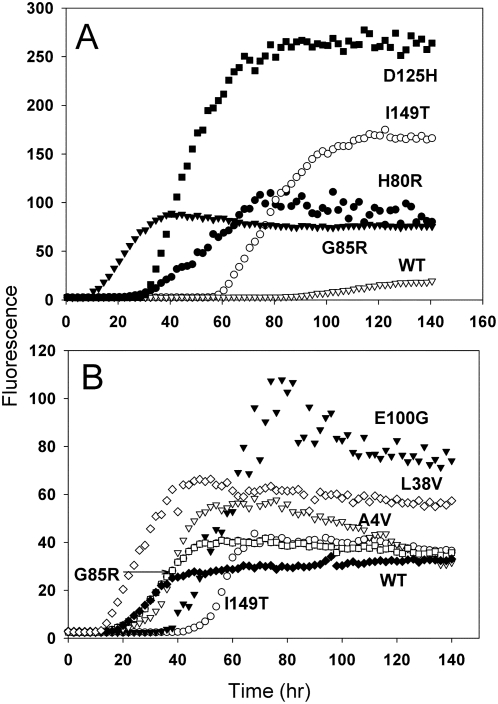
Amyloid formation from WT SOD1 and selected mutant SOD1 proteins at pH 5. SOD1 was incubated as in Figure 1 in 50 mM sodium acetate, 0.1 M NaCl, pH 5, plus 1 M guanidine hydrochloride (A) or 2 M guanidine hydrochloride (B). A: D125H (solid squares), I149T (open circles), H80R (solid circles), G85R (solid triangles), WT (open triangles). B: L38V (open diamonds), E100G (solid triangles), A4V (open triangles), I149T (open circles), G85R (open squares), WT (solid diamonds).

At pH 5, however, many ALS mutants showed enhanced amyloid formation compared to WT SOD1. Representative fluorescence time courses for selected mutants are shown in Figure 5, and a complete set of kinetic parameters is presented in Table 3. WT SOD1 forms barely detectable amounts of amyloid at this pH (Figure 1B). In 1 M guanidine, four out of five metal-binding-region mutants – D125H, G85R, H46R, and H80R (but not S134N) – in addition to the dimer interface mutant I149T, showed greater amplitudes of fluorescence change and shorter lag times than WT (Figure 5A and Table 3). In contrast, no enhancement was observed for the beta barrel mutants and the dimer interface mutant A4V at this guanidine concentration. In 2 M guanidine, on the other hand, all the mutants except S134N and D125H formed more amyloid than WT, with the greatest yield of amyloid observed for the “wild-type-like” beta barrel mutants E100G, G93A, and L38V (Figure 5B and Table 3). For the metal-binding-region mutants, the stimulation of amyloid formation at pH 5 is likely due to the fact that these mutations allow dissociation of metals from the enzyme at a higher pH than is observed for WT SOD1. For the wild-type-like mutations the stimulation of amyloid formation at pH 5 may be due to increased susceptibility of the mutants to acid-induced unfolding.

The enhancement of amyloid formation was manifested as a greater amplitude of ThT fluorescence and/or a reduced lag time. The amplitude of ThT fluorescence must be interpreted cautiously, and it is possible that some of the increase could be due to formation of an aggregate with a greater affinity for ThT, rather than a greater amount of aggregate. While mutants that showed both a greater fluorescence amplitude and a shorter lag time clearly enhance amyloid formation, those cases where the increased fluorescence was accompanied by a lag time equal to that of WT (e.g. L38V in 2 M urea) or even greater than that of WT (E100G in 2 M urea) are difficult to explain, and in these cases the evidence for an enhancing effect of the mutation on amyloid formation is not as strong.

### Effect of Mutations on Fibrillation of Apo-SOD1 at pH 7

Apoproteins with a variety of ALS mutations (A4V, C146R, E100G, G85R, H46R, and I113T) formed amyloid at pH 7 in the presence of 1 M guanidine; no consistent effect of the mutations was observed, with some mutations forming more amyloid and others less amyloid than WT SOD1. In the absence of guanidine most of the mutations appeared to reduce the yield of amyloid, with the A4V mutation completely abolishing amyloid formation (Table S1). However, electron microscopy of apo-A4V incubation mixtures revealed a variety of amorphous aggregates (data not shown), suggesting that A4V promoted the formation of amorphous aggregates instead of fibrillar aggregates. The possible existence of multiple aggregation pathways, some leading to amyloid and some to amorphous aggregates, both containing intermolecular disulfide bonds (Figure 3), make the effects of mutations on apo-SOD1 amyloid formation difficult to interpret.

**Figure 6:**
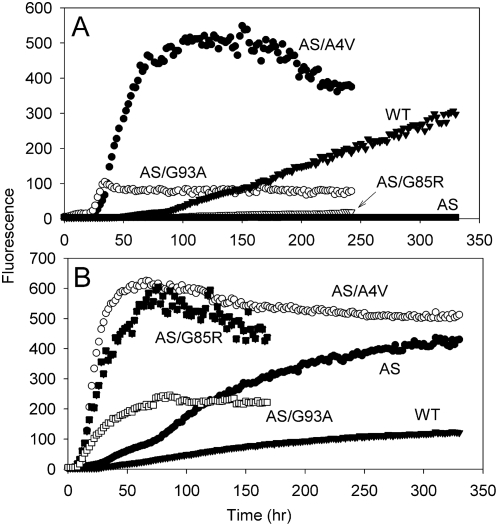
Aggregation assays with mutant SOD1 lacking free sulfhydryl groups. A) Apo-WT (solid triangles), apo-AS (solid squares), apo-AS/A4V (solid circles), apo-AS/G93A (open circles), and apo-AS/G85R (open triangles) were incubated in 50 mM MOPS, 0.1 M NaCl, 1 mM EDTA, pH 7, at 37°C. B) WT (solid inverted triangles), AS (solid circles), AS/A4V (open circles), AS/G93A (open squares), and AS/G85R (solid squares) were incubated in 50 mM MOPS, 0.1 M NaCl, 0.1 M TCEP, pH 7, at 37°C.

The situation was quite different for SOD1 mutants lacking free cysteine. No amyloid was observed for apo-AS (WT) SOD1 (Figure 6A), even after two weeks of incubation. On the other hand, apo-AS/A4V and AS/G93A formed amyloid readily, and a very small amount of amyloid was produced from apo-AS/G85R. These results indicate that when intermolecular disulfide formation is prevented by the absence of free cysteines, only metal-free SOD1 proteins bearing certain ALS mutations can form amyloid at neutral pH. Similar results were obtained by DiDonato et al. [16] for the incubation of AS SOD1 and SOD1 proteins bearing mutations in an AS background at pH 3.5.

### Effect of Mutations on Fibrillation of Metalated SOD1 in 0.1 M TCEP at pH 7

In the presence of 0.1 M TCEP, nearly all FALS mutants examined showed enhanced amyloid formation compared to WT SOD1, and in a number of cases (C146R, H46R, and H80R) the lag time was substantially reduced (Table 4).

**Table 4:** Kinetic parameters for amyloid formation from metalated SOD1 at pH 7 in the presence or absence of TCEP.

SOD1	no TCEP		0.1 M TCEP		Location	Cu	Zn	Sum
	amplitude	lag (hr)	amplitude	lag (hr)				
WT	---	---	118±19	33±6		0.48	3.06	3.54
G93A	---	---	305±93	26±3	BB	0.62	2.24	2.86
C146R	95±4	5±2	86±29	6±2	SS	0.74	2.58	3.32
A4V	---	---	294±141	34±12	DI	0.40	2.40	2.80
G85R	---	---	204±25	115±24	MBR	0.24	2.96	3.20
H46R	---	---	237±34	9±1	MBR	0.20	1.26	1.46
H80R	43±13	26±13	159±41	3±1	MBR	0.10	0.40	0.50
S134N	---	---	134±37	42±3	MBR	0.07	1.62	1.69
AS	---	---	412±177	30±11	MBR	0.46	3.06	3.52
AS/A4V	---	---	620±117	6±1	DI	0.36	2.12	2.48
AS/G93A	---	---	212±116	8±1	BB	1.00	1.48	2.48
AS/G85R	---	---	568±161	7±1	MBR	0.39	1.56	1.95

SOD1 was incubated in 50 mM MOPS, 0.1 M NaCl, pH 7 with or without TCEP. The time course of ThT fluorescence was fit to a sigmoidal equation and the amplitude and lag time determined as described in “Materials and Methods”. Dashes indicate that no amyloid was formed. Location: BB = beta barrel; DI = dimer interface; MBR = metal-binding region. Cu and Zn contents are metal atoms per dimer.

The ALS mutant C146R, which lacks the 57-146 intramolecular disulfide bond, produced a slightly lower yield of amyloid than WT SOD1, but the lag time was much shorter. The kinetics were similar whether or not TCEP was present, as would be expected, since TCEP should have no effect on a protein which lacks disulfide bonds. The result with C146R indicates that the absence of the intramolecular disulfide bond is sufficient to allow SOD1 to form amyloid. The only other SOD1 protein that formed amyloid at pH 7 even in the absence of either EDTA or TCEP was H80R, a mutant SOD1 which is nearly devoid of both copper and zinc (Table 4) and therefore behaves like other apo-SOD1 proteins (Figure 2).

In the presence of 0.1 M TCEP AS, AS/A4V, AS/G93A, and AS/G85R all showed a greater yield of amyloid formation relative to WT SOD1 (Figure 6B and Table 4). The lag time was much shorter than that of WT for AS/A4V, AS/G93A, and AS/G85R, but not for AS. The effect of removing cysteines 6 and 111 on the yield of amyloid, even in the absence of ALS mutations, is difficult to explain; the replacement of these two cysteines may either cause some destabilization of the tertiary structure of SOD1 [42] or promote the formation of amyloid instead of other types of aggregates, like amorphous aggregates.

## Discussion

The results presented here lead to three principal conclusions: 1) Metal-free SOD1^S-S^ and metalated SOD1^2SH^ self-assemble into amyloid fibrils at physiological temperature, pH and ionic strength. 2) Amyloid both with and without intermolecular disulfide bonds may be formed, depending on the incubation conditions. 3) ALS mutations can promote the assembly of SOD1 into amyloid by mechanisms which include facilitating the loss of metals and reduction of the intramolecular disulfide bond.

### SOD1 Forms Amyloid At Physiological pH, Ionic Strength and Temperature

This work demonstrates clearly that SOD1 forms fibrillar aggregates at physiological pH, ionic strength and temperature; these fibrils were identified as amyloid by a variety of ultrastructural and spectroscopic techniques. A previous study showed that amyloid formation occurred at pH 3.5 and was accelerated by the presence of EDTA, but no amyloid was observed at neutral pH [16]. The present work also shows amyloid formation at acidic pH but demonstrates further that either removal of copper and zinc by EDTA treatment or cleavage of the C7-C146 disulfide bond by concentrated TCEP was sufficient to trigger amyloid formation at pH 7. Amyloid formed faster in the presence of 1 M guanidine, but guanidine was not required. The stimulation of amyloid formation by a restricted range of guanidine concentrations, 0.5 to 2 M, suggests that metal loss or disulfide cleavage facilitates conversion of SOD1 to a partially unfolded form with an amyloidogenic conformation.

While this manuscript was in preparation, a study appeared demonstrating the formation of amyloid from SOD1 lacking both disulfide bonds and metals (apo-SOD1^2SH^), but not from metal-free SOD1 with its disulfide bond intact (apo-SOD1^S-S^) [13]. The reason for this discrepancy with our results is unknown, but it is possible that: 1) in the other study the incubations were not conducted long enough to observe amyloid (note the long lag time in Figure 2A), 2) the use of a lower rate of agitation (150 rpm vs. 960 rpm) in the other study made amyloid formation inefficient, and 3) the use of Teflon balls in the present study provided hydrophobic surfaces that promoted aggregation.

The production of amyloid required agitation of the microwell plates and inclusion of a Teflon sphere in each well. Agitation in the presence of Teflon has frequently been used as a tool to accelerate amyloid formation from bona fide amyloidogenic proteins whose aggregation requires weeks or months when such agitation is not used. For example, wild-type α-synuclein, which is the main constituent of the Lewy bodies found in Parkinson's disease, took between three and nine weeks to form fibrils when incubated at 37°C without agitation [43], but fibrillation was complete after 36–48 hr when the solutions were agitated with a Teflon stir bar or with a Teflon sphere in a microplate [28], [44]. Explanations proposed for effects of agitation include increased exposure of hydrophobic groups at an air-water interface [45] or on Teflon surfaces [37], or increased fragmentation of fibrils and creation of more seeds to nucleate fibril formation. Teflon has been suggested to act as a sorbent surface that mimics the nonpolar interior of a lipid bilayer [37]. Since recent studies have demonstrated the presence of SOD1 bound to mitochondrial membranes [46]–[47] and demonstrated a change in secondary structure of SOD1 upon binding to phospholipid vesicles [48], it is possible that membranes seed the formation of SOD1 aggregates *in vivo*.

### SOD1 Can Form Amyloid With or Without Intermolecular Disulfide Bonds

Recently there has been some controversy about the role of intermolecular disulfide bonds in SOD1 aggregation. While disulfide-linked SOD1 aggregates have been found in transgenic mice expressing ALS-related mutant SOD1 [49]–[50], and while there is some evidence that intermolecular disulfide formation plays a major role in SOD1 aggregation in transfected cultured cells [51]–[52], other work has shown that transfected cells expressing SOD1 variants lacking cysteine also develop SOD1 aggregates [42], [53], [54]. Our results demonstrate that amyloid formation *in vitro* is not necessarily accompanied by the formation of intermolecular disulfide bonds. At neutral pH, apo-AS SOD1, which lacks free cysteines, is incapable of forming amyloid, but the presence of ALS mutations confers a tendency to form amyloid even when free cysteines are absent (Figure 6A). DiDonato et al.[16] observed similar behavior for AS SOD1 and ALS mutants in an AS background for incubations at pH 3.5. Also, Banci et al. [17]–[18] reported that extended incubation of apo-SOD1 led to large oligomeric aggregates containing disulfide bonds and that ALS mutations generally led to faster oligomerization, while apo-AS-SOD1 did not aggregate. They did not report the properties of AS-SOD1 bearing ALS mutations.

Furthermore, a wide variety of SOD1 proteins, both containing and lacking free cysteines, and both with and without ALS mutations, formed amyloid in the presence of high concentrations of TCEP (Figures 2B and 6B and Table 4). In summary, we have found that amyloid both with and without intermolecular disulfide bonds may be formed, depending on the incubation conditions (Figure 3) and thus that the occurrence of amyloid-like aggregates *per se* does not depend on forming disulfide bonds.

### ALS Mutations Promote Amyloid Formation

The results suggest that ALS mutations may enhance amyloid formation by facilitating the removal of metals from SOD1 and by increasing the susceptibility of SOD1 to reduction of its intramolecular disulfide bond.

### ALS Mutations Promote Amyloid Formation by Facilitating Loss of Metals

ALS mutations promote amyloid formation by allowing dissociation of metals from SOD1 at pH values where metals remain tightly bound to WT SOD1. Thus ALS mutations led to enhanced amyloid formation at pH 5, and the effect was most pronounced with metal-binding-region mutants (Figure 5, Table 3). A previous study demonstrated that several metal-binding-region mutants display increased sensitivity of zinc binding to low pH, with the pK_a_ for the loss of zinc binding shifted from 3.8 for the wild type protein to higher values ranging from 4.7 to 7.3 [55]. In the experiments shown in Table 3, H46R, whose pKa for the loss of zinc is 6.0 [55], formed amyloid in high yield with a short lag time at pH 5. Also, H80R, which is nearly devoid of metals, showed a similar yield of amyloid in 1 M guanidine at pH 5 as at pH 3, albeit with a longer lag time. The metal-binding region mutants G85R and D125H also produced a significantly greater yield of amyloid at pH 5 and 1 M guanidine than WT SOD1; the zinc-binding properties of these mutants have not been reported. Also, several wild-type-like mutants, including A4V and L38V, were shown previously to have lower zinc affinities than the wild type protein in the presence of 2 M urea [56]. In a physiological context, loss of metals could be promoted by a local acidic environment or by other cellular stresses, and this would occur more readily with mutant SOD1. Alternatively, newly synthesized mutant SOD1 proteins may form amyloid before metals are coordinated. It should be noted that the failure of S134N, also a metal-binding region mutant, to form any amyloid at pH 5 is difficult to explain (see also below).

### ALS Mutations Promote Amyloid Formation by Facilitating Partial Unfolding or Monomerization of Apo-SOD1

The presence of many wild-type-like ALS mutations leads to reduced stability of apo-SOD1 against thermal or guanidine-induced denaturation [57]–[61]. Hence the effect of the A4V and G93A mutations in potentiating amyloid formation from apo-AS SOD1 (Figure 6) indicates that apo-AS SOD1 bearing either of these mutations undergoes partial unfolding under conditions where the corresponding protein lacking ALS mutations remains stably folded. The amount of amyloid formation is correlated with the effect of the mutations on stability: A4V, one of the most highly destabilized ALS mutants, forms amyloid in very high yield, with less amyloid formed from the moderately destabilized G93A, and G85R, the least destabilized of the three mutants, forming virtually no amyloid. It should be noted that destabilization of apo-SOD1 is not observed for most of the metal-binding-region mutants that have been examined. [59].

Evidence has also been presented that dimer dissociation is an obligatory step in SOD1 aggregation [62]–[64]. While apo-SOD1 is predominantly dimeric, loss of metals makes dimers more susceptible to dissociation by acidic pH [65], detergents [66], or guanidine [14]. Studies of the guanidine-induced unfolding of the holo-dimer of SOD1 suggested that the E100G, G85R and G93A mutations had little effect on dimer dissociation [61], while some weakening of the dimer interface of apo-SOD1 was observed with the E100G and G85R mutations, and especially with the dimer interface I113T mutation [60].

Thus the published studies support the idea that various factors may contribute to the enhancing effect of ALS mutations on amyloid formation at acidic pH: Metal-binding-region mutations may facilitate release of bound metals; both metal-binding-region and dimer-interface mutants may enhance dimer dissociation; and wild-type-like mutations in both the beta barrel and the dimer interface may facilitate the unfolding of apo-SOD1. The close linkage between dimer dissociation, demetalation and unfolding make it difficult to ascribe a stimulation of amyloid formation to an effect on just one of these three processes.

### ALS Mutations Promote Amyloid Formation by Increasing the Susceptibility of SOD1 to Disulfide Reduction and by Destabilizing Disulfide-Reduced SOD1

The present results show that loss of the disulfide bond linking the zinc loop to the beta barrel as a result of treatment with TCEP allows conversion of SOD1 to a form capable of forming amyloid. We have no data concerning the metal content of the proteins after TCEP treatment, and it is possible that some loss of metals occurred during these incubations. Apo-WT SOD1 in fact formed amyloid with a lag time of only a few hours in 0.1 M TCEP; under these conditions, apo-G93A showed a time course very similar to that of WT SOD1, while two metal-binding-region mutant SOD1 proteins formed much less amyloid (data not shown; see [67]). Additionally, although previous work showed that the 57-146 disulfide bond is reduced faster in many mutant proteins than in wild type SOD1, we did not measure the extent of disulfide reduction in these experiments. Hence, it is possible that in some cases a release of metals as a result of the mutation was at least partly responsible for the effect of the mutation and not just an increased rate of disulfide reduction.

However, it should be noted that WT SOD1^2SH^ retains both copper and zinc [68]–[69]. Furthermore, C146R, which contains 0.74 copper and 2.58 zincs per dimer, formed amyloid upon incubation at pH 7 in the absence of TCEP with the same kinetics as observed in the presence of TCEP. This finding supports the idea that cleavage of the disulfide bond is sufficient to promote amyloid formation from metalated SOD1 and that ALS mutations promote formation of amyloid from metalated proteins whose disulfide bonds have been reduced. Nearly all the ALS mutant forms of SOD1 that we examined have a significantly greater tendency to form amyloid upon incubation with TCEP than WT SOD1. This is due, at least in part, to an increased susceptibility of the 57-146 disulfide bond to reduction in many mutant proteins, including A4V, G93A, and G85R, as demonstrated previously [30].

Once reduction has occurred, mutant SOD1^2SH^ may also more readily undergo dissociation to monomers or unfolding than the wild-type protein. Regarding dimer dissociation, a number of groups have shown that reduction of the disulfide bond causes apo-SOD1 to dissociate into monomers [14], [15], [70]. Furthermore, while for the wild-type protein metalated SOD1^2SH^ remains dimeric [14], [15], several ALS mutations, including A4V, G85R, G93A, H46R and S134N, were found to weaken the dimer interface of metalated SOD1 [30].

Regarding unfolding, the A4V and G93A mutations have been shown to destabilize both metal-free and zinc-containing SOD1 whose intramolecular disulfide bond is reduced [71]. The fact that S134N formed amyloid with a yield and lag time not significantly different from WT is unexpected in view of the enhanced accessibility of the cysteines in that mutant to alkylation [30], but it should be noted that the S134N mutation is one of the metal-binding-region mutants that does not destabilize apo-SOD1^S-S^ [59], and its effect on the unfolding of either metalated or metal-free SOD1^2SH^ has not been examined. It is possible that the S134N mutation, along with certain others that do not appear to destabilize SOD1 (e.g. D101N [59]), cause ALS by a mechanism that does not involve amyloid formation or other forms of aggregation.

### Effect of Nonpolar Solvents

At acidic pH acetonitrile, but not trifluoroethanol, promoted the formation of amyloid. Acetonitrile-induced stabilization of the beta pleated sheet conformation of amyloidogenic proteins has been observed previously [72]–[80], and in several cases acetonitrile was observed to stabilize beta sheet specifically, while trifluoroethanol or hexafluoroisopropanol favored alpha helices [75]–[77], [81], in line with the present results.

### Relevance of In Vitro Amyloid Formation to FALS

The findings presented here may be highly relevant to the process by which SOD1 aggregation is triggered in ALS. While the presence of amyloid in ALS neurons has not been directly demonstrated, fibrillar inclusions containing SOD1 occur in neurons of FALS patients, and fibrillar inclusions containing SOD1 that bind amyloid-specific dyes have been observed in certain transgenic mouse models of FALS. (See the “Introduction.”)

Our data show enhanced amyloid formation upon removal of metals from SOD1 or reduction of the intramolecular disulfide bond. A variety of studies have shown that both undermetalated and disulfide-reduced SOD1 exist *in vivo* and that their presence is exacerbated by the presence of mutations related to ALS. A significant fraction of WT SOD1 is incompletely metalated *in vivo* [82]–[85], and both incompletely metalated and disulfide-reduced SOD1 are present in transgenic mice expressing various mutants of SOD1 [86]–[87]. After translation by the ribosome, SOD1 binds zinc by a process which has not been characterized; then the binding of copper and concomitant formation of the intramolecular disulfide bond are catalyzed by the copper chaperone for SOD1 (CCS) [88]. An ALS mutation may accelerate the loss of metals from metalated SOD1 or the reduction of the disulfide bond of SOD1, leading to the production of amyloid. Alternatively, the presence of the mutation may make SOD1 a poorer substrate for CCS, thus prolonging the lifetime of the amyloidogenic form of SOD1.

Our results also raise the possibility that SOD1 aggregates associated with ALS may be heterogeneous in nature, varying in regard to the presence or absence of both intramolecular and intermolecular disulfide bonds and in regard to metal content. As noted earlier, there is evidence for the formation in cells of aggregates both with and without disulfide bonds.

The *in vitro* aggregation system described in this study may be of use in further investigations of the molecular basis of ALS. For example, in both ALS and other diseases related to protein aggregation, there is considerable evidence that it is oligomeric aggregates, possibly including intermediates on the route to forming large insoluble aggregates, that are the toxic species which causes the disease, rather than the large aggregates themselves [89], [90]. Work is now in progress to examine the time course of changes in protein size and conformation under various conditions that favor amyloid formation and to identify and characterize intermediates in the aggregation process.

## Materials and Methods

### Chemicals

All chemicals were reagent grade. A neutralized solution of tris(2-carboxyethyl)phosphine (Bond-Breaker™ TCEP Solution) was purchased from Pierce Chemical Company. Mal-PEG, polyethylene glycol of molecular weight 5000 coupled to maleimide, was purchased from Nektar Pharmaceuticals (mPEG-MAL).

### SOD1 Expression and Purification

YEp351 expression vectors encoding both wild-type and mutant SOD1 proteins were introduced into *Saccharomyces cerevisiae*, and protein was expressed and purified as described previously [91]. Expression vectors encoding the C6A/C111S/G93A (AS/G93A) and C6A/C111S/G85R (AS/G85R) mutants were derived from the expression vector for the AS (C6A/C111S) mutant by introducing appropriate point mutations with the QuikChange Mutagenesis Kit (Stratagene). The identity of each purified protein was confirmed by determining its molecular weight with a Perkin Elmer Sciex API III triple quadrupole electrospray ionization mass spectrometer. In all cases a single peak with the expected molecular weight was observed. Apo-SOD1 was prepared by extensive dialysis of SOD1 against 0.1 M sodium acetate, 10 mM EDTA, pH 3.8 [55], followed by dialysis against 2.25 mM potassium phosphate, 1 mM EDTA, pH 7, for storage. All glassware and plasticware were soaked in 2% nitric acid before use.

### Metal Analysis

The copper and zinc content of each protein preparation was determined with a Perkin-Elmer 6100DRC inductively-coupled plasma mass spectrometer (ICP-MS). Purified protein samples were dialyzed vs. 2 mM sodium phosphate, pH 7. They were then diluted with metal-free 1 M nitric acid and analyzed along with a sample of the dialysis buffer as a blank, as well as standard copper and zinc solutions. A standardized quantity of gallium was added to all samples as an internal standard.

### Detection of Free Sulfhydryl Groups with Mal-PEG

Mal-PEG was dissolved in cold water to a concentration of 15 mM and immediately added to a protein solution for final concentrations of 0.5 mg/ml protein, 50 mM 3-(N-morpholino)propanesulfonic acid (MOPS), pH 7, 0.1 M NaCl, 3 mM Mal-PEG, 1% SDS. The mixture was incubated 3 hr at 37°C. The reaction was terminated by boiling in SDS sample buffer.

### Fluorescence Assay for Amyloid Formation

Amyloid formation was monitored by following the increase in the fluorescence of ThT [26]–[27]. A microplate assay similar to one described previously [33] was employed. Incubation buffers contained 50 mM citrate pH 3, formate pH 4, acetate pH 5, succinate pH 6, or MOPS pH 7, plus 0.1 M NaCl. Forty-µl samples containing 1 mg/ml SOD1 (65 µM polypeptide chain or 33 µM dimer) in buffers containing 10 µM ThT were pipetted into wells of a Corning Costar 384-well microplate with transparent bottoms, white walls, and non-binding surface (Corning 3653). A 3/32-inch Teflon ball (McMaster-Carr, Los Angeles) was placed in each well, and the plates were sealed with ThermalSeal plate sealers (Excel Scientific). The plates were incubated in a Thermo Labsystems Fluoroskan FL fluorescence microplate reader at either 24°C or 37°C with agitation at 960 rpm, and fluorescence readings were acquired every 30 minutes with excitation at 444 nm and emission at 485 nm. Quadruplicate samples were analyzed for each set of conditions.

The kinetic data (fluorescence (*F*) vs. time (*t*)) were fit to a sigmoidal equation using Sigmaplot:
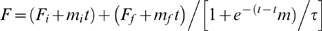
The initial baseline is *F_i_+m_i_t*, the final baseline in the plateau region is *F_f_+m_f_t*, and *t_m_* is the time to 50% of maximal fluorescence increase. The following kinetic parameters were then calculated: the lag time is given by *t_m_−2τ*, and the amplitude, *amp*, by *F_f_*−*F_i_* (*cf.* [28])_._

#### Spectra

Soluble SOD1 was dissolved in 2 mM sodium phosphate, pH 7. Amyloid was prepared by incubating SOD1 for 3 days in 10 mM sodium citrate, 0.1 M NaCl, pH 3, containing either 1 M guanidine or 20% acetonitrile, then pelleting the protein and resuspending it in 2 mM phosphate, pH 7. Absorbance spectra for detecting Congo Red binding were measured on a Shimadzu UV-2401PC spectrophotometer. Fluorescence spectra of anilinonaphthalene sulfonic acid (ANS) were collected with a Jobin-Yvon Fluoromax II spectrofluorometer using an excitation wavelength of 370 nm and emission wavelengths of 380–650 nm. Solutions contained 100 µM ANS and either 0 or 5 µM protein in 50 mM MOPS, 0.1 M NaCl, pH 7.

Circular dichroism spectra were acquired with a Jasco J-810 spectropolarimeter, using cylindrical cuvettes with a path length of 0.2 mm. Infrared spectra were acquired on a Nicolet 800 FTIR spectrometer by the attenuated total reflectance method. Solutions of SOD1 at a concentration of 0.3–0.5 mg/ml were deposited on the surface of a germanium prism and dried to form a thin film, and spectra were acquired.

### Electron Microscopy

Samples were diluted to 0.1 mg/ml, and 10-µl aliquots were applied to 300-mesh carbon-coated copper grids with formvar films (EM Sciences) which had been subjected to glow discharge. Samples were allowed to adsorb for 30–60 seconds; the grids were then blotted dry and treated for 30–60 seconds with 2% uranyl acetate. The grids were examined in a JEOL 1200EX-II electron microscope operated at 80 kV.

### Atomic Force Microscopy

A 10-µL aliquot of the protein solution was deposited on freshly cleaved mica. Excess water was wicked away using a small piece of absorbent paper and the sample was placed in a desiccator to dry. Humidity was controlled by placing the microscope under a low flow of dry nitrogen gas.

Images were acquired at ambient temperature with a Nanoscope IIIa Multimode scanning probe microscope (Digital Instruments, Santa Barbara, CA) using tapping mode. Rotated tip etched silicon probes with the J scanning head were employed. Scanning parameters varied with individual tips and samples, but typical ranges were as follows: tapping frequency, 300–400 kHz; driving amplitude, 65–75 mV; and scan rate, 0.5–2 Hz. Height and phase data were simultaneously collected using a Digital Instrument extender phase module. Acquired images were first plane-fitted and carefully flattened for the analysis.

### Polarization Microscopy

Congo Red was added to suspensions of SOD1 amyloid to give final concentrations of 0.3 mg/ml (19 µM) SOD1 and 45 µM Congo Red. One hundred µl was pipetted onto a microscope slide and allowed to dry. Excess Congo Red was removed by washing the slide with ethanol. The slide was examined in an Olympus BX-P transmitted light polarizing microscope outfitted with a 4-megapixel digital camera.

## Supporting Information

Text S1 Remetalation of SOD1
(0.03 MB DOC)

Text S2 Spectroscopic Characterization of Amyloid
(0.02 MB DOC)

Figure S1 Mal-PEG reactivity of as-isolated and remetalated preparations. For remetalation, apo-proteins in 0.1 M sodium acetate, pH 5.5, were incubated overnight on ice with 2 equivalents of ZnSO4 per dimer; then 0.5 equivalents of CuSO4 were added at 2-hr intervals. Protein samples were reacted with Mal-PEG as desribed in Materials and Methods. Lane 1, WT; lane 2, remetalated WT; lane 3, A4V; lane 4, remetalated A4V; lane 5, remetalated AS. While as-isolated WT and A4V contained predominantly protein with one or two free cysteines per chain, remetalated WT and A4V showed predominantly protein lacking free cysteines. As expected, the AS mutant has no free cysteines. Since SOD1 is not completely unfolded in 1% SDS, some of the buried cysteines at position 6 may not have completely reacted.
(3.08 MB TIF)

Figure S2 Atomic force microscopy of SOD1 amyloid. Amyloid was formed by incubating SOD1 in 1 M guanidine, pH 3, at 37°C. The right-hand image is the phase image. The field of each image is a 10 µM×10 µM square.
(1.98 MB TIF)

Figure S3 Electron microscopy of amyloid formed from apo- and reduced SOD1. Apo-SOD1 was incubated in 50 mM citrate, 0.1 M NaCl, 1 M guanidine, pH 3 (A) or in 50 mM MOPS, 0.1 M NaCl, 1 mM EDTA, pH 7, with (B–D) or without (E–G) 1 M guanidine. Metalated SOD1 was incubated in 50 mM MOPS, 0.1 M NaCl, pH 7, with 1 M guanidine and 10 mM TCEP (H–I) or 0.1 M TCEP (without guanidine) (J–L). Samples are WT SOD1 (A,B,C,D,G,H,J,K,L), E100G (E), I113T (F), or C146R (I). Amyloid formed from apo-SOD1 in 1 M guanidine at pH 3 (A) contained fibrils similar to those seen with metalated SOD1. At pH 7 and 1 M guanidine, apo-SOD1 formed mostly short fibrils which were not as highly clumped as those formed at pH 3, along with some spherical aggregates (B–C), although long thinner fibrils with diameters of 3–5 nm were sometimes observed (D). Both short fibrils and mesh-like networks were observed with both apo-SOD1 incubated at pH 7 in the absence of guanidine (E–G) and TCEP-treated SOD1, both in the presence (H–I) or absence (J–L) of guanidine. Spherical structures were present with amyloid prepared from apo-SOD1 but not with amyloid prepared from TCEP-treated SOD1.
(6.26 MB TIF)

Figure S4 Dye-binding and spectroscopic properties of SOD1 amyloid. A. SOD1 amyloid binds Congo Red. SOD1 amyloid was prepared by incubation for 3 days at 37°C at pH 3 and 1 M guanidine as in Figure 1 and collected by pelleting. Spectra are 5 µM Congo Red (solid line), 5 µM Congo Red plus 10 µM SOD (dash line), and the difference spectrum (dot-dash line). SOD1 binding caused a red shift in the absorbance with a maximum in the difference spectrum at 542–543 nm. B. Congo Red birefringence of SOD1 amyloid. Amyloid was prepared as in Figure 1 and examined in the presence of Congo Red in a polarization microscope. Left and right, without and with cross-polarization. The field of each image is 100 µm×85 µm. C. Circular dichroism of soluble SOD1 and SOD1 amyloid. Spectra are soluble SOD1 (solid line), amyloid prepared from metalated SOD1 at pH 3 in 1 M guanidine (dash line) or 20% acetonitrile (dot-dash line). D. Fourier transform infrared spectra of soluble SOD1 and SOD1 amyloid. Spectra of soluble SOD1 (open inverted triangles), amyloid formed from metalated SOD1 at pH 3 in 1 M guanidine (open diamons) or 20% acetonitrile (solid squares), from apo-AS/A4V SOD1 at pH 7 in 1 M guanidine (solid circles), or from metalated AS/A4V SOD1 in 0.1 M TCEP (solid triangles).
(1.71 MB TIF)

Table S1 Kinetic Parameters for Amyloid Formation from Apo-SOD1 proteins
(0.02 MB DOC)
