# Supplementary material for: Loss of Metal Ions, Disulfide Reduction and Mutations Related to Familial ALS Promote Formation of Amyloid-Like Aggregates from Superoxide Dismutase
Source: PLoS One. 2009 Mar 27;4(3):e5004. doi: 10.1371/journal.pone.0005004 (PMC2659422; doi:10.1371/journal.pone.0005004)
Supplement: Text S1 — Remetalation of SOD1 (0.03 MB DOC) [file pone.0005004.s001.doc]

**Text S1. Remetalation of SOD1**

As stated in the main text, when we attempted to use a published procedure for remetalating apo-SOD1, we failed to obtain either fully metalated or fully active SOD1 proteins. Previous studies have shown that use of standard metalation procedures with mutant SOD1 proteins isolated from a yeast expression system often led to binding of either copper or zinc to the incorrect site [23,24].

We explored other procedures for remetalation, some of which involved adding a full complement of zinc and copper to apo-proteins, and others in which we attempted to restore a full complement of metals to the as-isolated preparations which are partially metalated to begin with. We did succeed in finding remetalation conditions that led to some protein preparations with approximately two zincs and two coppers per dimer and full enzymatic activity. However, when incubated under conditions that promoted amyloid formation from the as-isolated preparations (see below), these remetalated preparations failed to form any species capable of binding thioflavin T (data not shown). By contrast, use of the same procedures to restore the missing metals to the mutants lacking free cysteines, AS and AS/A4V, gave proteins that were not only fully metalated and fully active but also had aggregation kinetics indistinguishable from the as-isolated proteins (data not shown). This raised the possibility that exposure to free Cu^2+^ ion during the remetalation had led to oxidation of free cysteines in the proteins that contained cysteines at positions 6 and 111. Loss of reduced cysteines was confirmed by the failure of these remetalated preparations to react with maleimido-polyethylene glycol (Mal-PEG) [92,30] (Figure S1). The apparent oxidation of cysteine appeared to be an irreversible conversion to a sulfinic or sulfonic acid, since Mal-PEG reactivity was not restored by extended treatment of the remetalated preparations with DTT or TCEP (data not shown), a treatment which should reduce disulfide bonds or sulfenic acids [29-30]. Previous studies have shown that cysteine 111, the solvent accessible cysteine of SOD1, is oxidized to the sulfonic acid upon treatment of SOD1 with 1 mM CuCl_2_ at room temperature for 20 minutes [93].

References not cited in the main text:

92. Makmura L, Hamann M, Areopagita A, Furuta S, Munoz A et al. (2001) Development of a sensitive assay to detect reversibly oxidized protein cysteine sulfhydryl groups. Antioxid Redox Signal 3: 1105-18.

93. Fujiwara N, Nakano M, Kato S, Yoshihara D, Ookawara T et al. (2007) Oxidative modification to cysteine sulfonic acid of Cys111 in human copper-zinc superoxide dismutase. J Biol Chem 282: 35933-44.
