# Supplementary material for: Loss of Metal Ions, Disulfide Reduction and Mutations Related to Familial ALS Promote Formation of Amyloid-Like Aggregates from Superoxide Dismutase
Source: PLoS One. 2009 Mar 27;4(3):e5004. doi: 10.1371/journal.pone.0005004 (PMC2659422; doi:10.1371/journal.pone.0005004)
Supplement: Text S2 — Spectroscopic Characterization of Amyloid (0.02 MB DOC) [file pone.0005004.s002.doc]

**Text S2. Spectroscopic Characterization of Amyloid**

The amyloid character of the aggregated material was verified by a number of spectroscopic methods. The pelletable material produced during incubation in 1 M guanidine at pH 3 bound the amyloid-specific dye Congo red, as judged by a shift in the absorbance maximum from about 503 to 535 nm with a maximum in the difference spectrum at 542-543 nm (Figure S3A) [31-32], as well as a green birefringence (Figure S3B) [33-34]. The trough in the circular dichroism (CD) spectrum shifted from 209 nm for soluble SOD1 to 220-222 nm for pelletable material formed at pH 3 in the presence of either guanidine or acetonitrile (Figure S3C), and a similar spectrum was obtained for amyloid formed from apo-SOD1 at pH 7 and 1 M guanidine (data not shown). This spectrum is characteristic of amyloid and differs from that of soluble beta-sheet proteins, where the trough usually occurs at about 215 nm [35-40]. No CD signal was observed for amyloid prepared from either apo-SOD1 at pH 7 in the absence of guanidine or from SOD1 incubated in 0.1 M TCEP, probably as a result of a loss of transmitted light caused by scattering from large particles.

FTIR spectra showed a shift in the maximum from 1644 cm^-1^ to 1631 cm^-1^ upon amyloid formation (Figure S3D), attributable to a significant increase and/or reorganization of β-sheet [41]. Similar spectra were obtained for amyloid prepared from both metalated and apo-SOD1 under a variety of incubation conditions. This suggests that amyloid produced under different sets of conditions have a common structural organization.

Fluorescence spectra obtained with an excitation wavelength of 290 nm showed a shift of the emission maximum from 353 nm to 339 nm upon amyloid formation (data not shown). This suggests that W32, the sole tryptophan residue, which extends out from the surface of the β-barrel on the side opposite the metal-binding region, moves to a nonpolar environment, probably buried in the interface between adjacent SOD1 molecules in the amyloid fibril. Addition of SOD1 amyloid to a solution of anilinonaphthalene sulfonic acid produced only a small (~20%) enhancement of fluorescence (data not shown), indicating that there is very little solvent exposure of hydrophobic clusters in SOD1 amyloid [94].

Reference not cited in the main text:

94. Goto Y, Fink AL (1989) Conformational states of beta-lactamase: molten-globule states at acidic and alkaline pH with high salt. Biochemistry 28, 945-952.
