# Supplementary figures and images for: Loss of Metal Ions, Disulfide Reduction and Mutations Related to Familial ALS Promote Formation of Amyloid-Like Aggregates from Superoxide Dismutase
Source: PLoS One. 2009 Mar 27;4(3):e5004. doi: 10.1371/journal.pone.0005004 (PMC2659422; doi:10.1371/journal.pone.0005004)

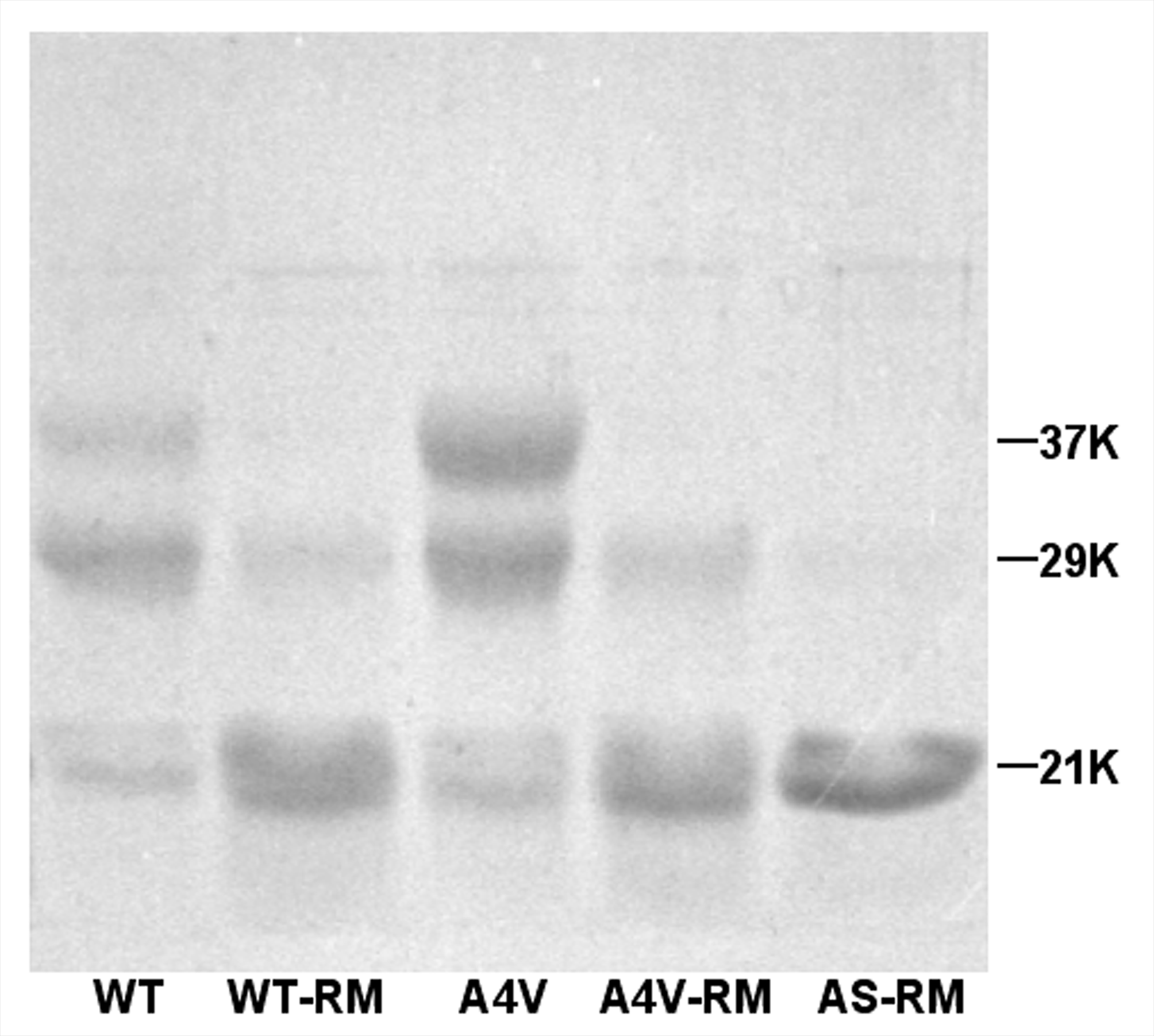

Supplement: Figure S1 — Mal-PEG reactivity of as-isolated and remetalated preparations. For remetalation, apo-proteins in 0.1 M sodium acetate, pH 5.5, were incubated overnight on ice with 2 equivalents of ZnSO4 per dimer; then 0.5 equivalents of CuSO4 were added at 2-hr intervals. Protein samples were reacted with Mal-PEG as desribed in Materials and Methods. Lane 1, WT; lane 2, remetalated WT; lane 3, A4V; lane 4, remetalated A4V; lane 5, remetalated AS. While as-isolated WT and A4V contained predominantly protein with one or two free cysteines per chain, remetalated WT and A4V showed predominantly protein lacking free cysteines. As expected, the AS mutant has no free cysteines. Since SOD1 is not completely unfolded in 1% SDS, some of the buried cysteines at position 6 may not have completely reacted. (3.08 MB TIF) [file pone.0005004.s003.tif]

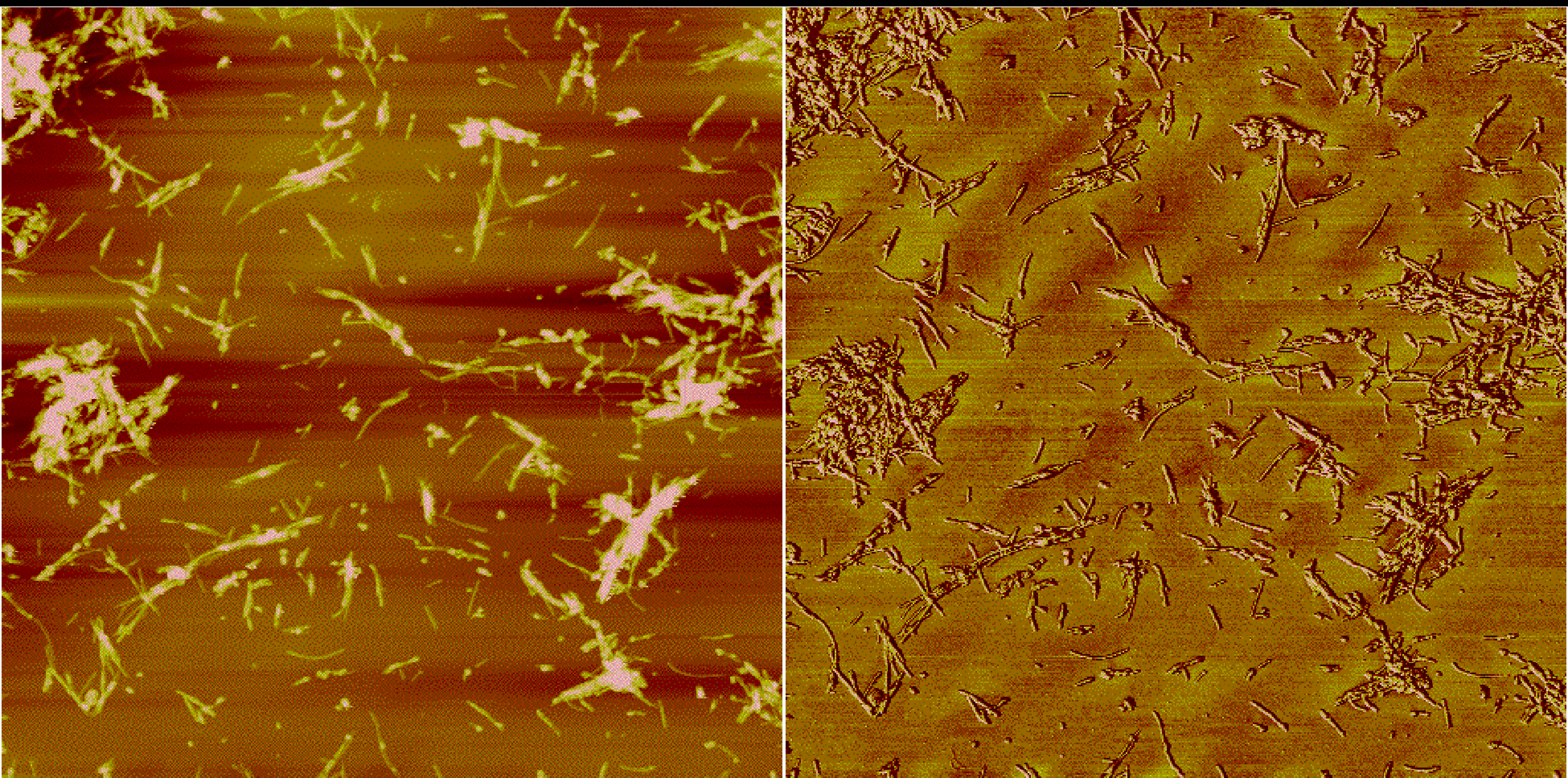

Supplement: Figure S2 — Atomic force microscopy of SOD1 amyloid. Amyloid was formed by incubating SOD1 in 1 M guanidine, pH 3, at 37°C. The right-hand image is the phase image. The field of each image is a 10 µM×10 µM square. (1.98 MB TIF) [file pone.0005004.s004.tif]

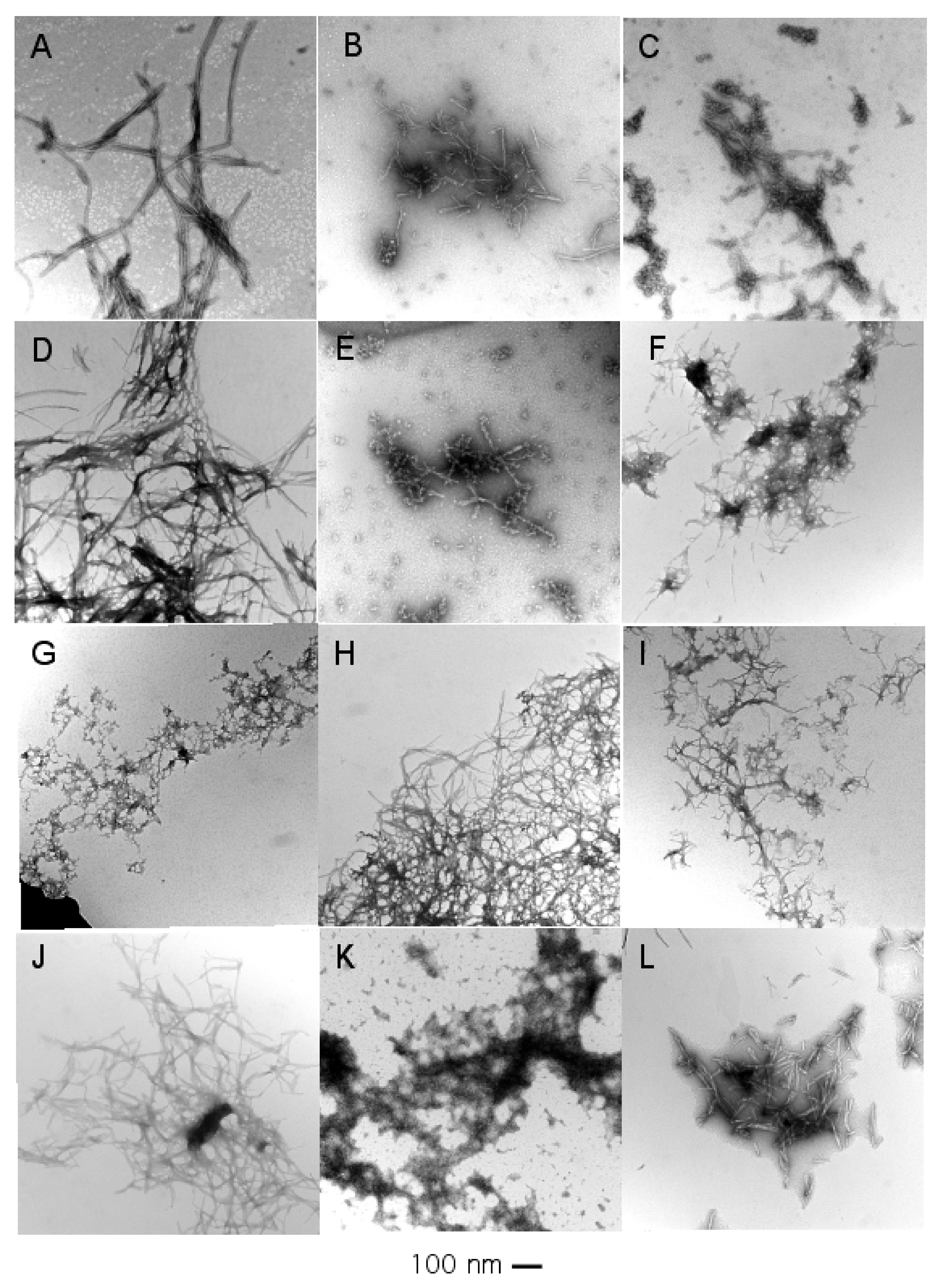

Supplement: Figure S3 — Electron microscopy of amyloid formed from apo- and reduced SOD1. Apo-SOD1 was incubated in 50 mM citrate, 0.1 M NaCl, 1 M guanidine, pH 3 (A) or in 50 mM MOPS, 0.1 M NaCl, 1 mM EDTA, pH 7, with (B–D) or without (E–G) 1 M guanidine. Metalated SOD1 was incubated in 50 mM MOPS, 0.1 M NaCl, pH 7, with 1 M guanidine and 10 mM TCEP (H–I) or 0.1 M TCEP (without guanidine) (J–L). Samples are WT SOD1 (A,B,C,D,G,H,J,K,L), E100G (E), I113T (F), or C146R (I). Amyloid formed from apo-SOD1 in 1 M guanidine at pH 3 (A) contained fibrils similar to those seen with metalated SOD1. At pH 7 and 1 M guanidine, apo-SOD1 formed mostly short fibrils which were not as highly clumped as those formed at pH 3, along with some spherical aggregates (B–C), although long thinner fibrils with diameters of 3–5 nm were sometimes observed (D). Both short fibrils and mesh-like networks were observed with both apo-SOD1 incubated at pH 7 in the absence of guanidine (E–G) and TCEP-treated SOD1, both in the presence (H–I) or absence (J–L) of guanidine. Spherical structures were present with amyloid prepared from apo-SOD1 but not with amyloid prepared from TCEP-treated SOD1. (6.26 MB TIF) [file pone.0005004.s005.tif]
