# Supplementary material for: Loss of Metal Ions, Disulfide Reduction and Mutations Related to Familial ALS Promote Formation of Amyloid-Like Aggregates from Superoxide Dismutase
Source: PLoS One. 2009 Mar 27;4(3):e5004. doi: 10.1371/journal.pone.0005004 (PMC2659422; doi:10.1371/journal.pone.0005004)
Supplement: Table S1 — Kinetic Parameters for Amyloid Formation from Apo-SOD1 proteins (0.02 MB DOC) [file pone.0005004.s007.doc]

# Table S1. Kinetic Parameters for Amyloid Formation from Apo-SOD1 proteins

1 M guanidine no guanidine

| Apo-SOD1 | Amplitude | Lag (hr) | Amplitude | Lag (hr) |
| --- | --- | --- | --- | --- |
| WT | 248 ± 109 | 26 ± 8 | 294 ± 94 | 52 ± 11 |
| E100G | 498 ± 141 | 23 ± 1 | 179 ± 84 | 62 ± 16 |
| C146R | 521 ± 292 | 13 ± 4 | 76 ± 12 | 23 ± 6 |
| A4V | 203 ± 3 | 17 ± 2 | ---- | ---- |
| I113T | 91 ± 22 | 27 ± 6 | 99 ± 58 | 41 ± 3 |
| G85R | 285 ± 145 | 62 ± 1 | 125 ± 34 | 43 ± 14 |
| H46R | 73 ± 11 | 60 ± 1 | 138 ± 57 | 56 ± 5 |
| AS |  |  | ---- | ---- |
| AS/A4V |  |  | 554 ± 39 | 29 ± 10 |
| AS/G93A |  |  | 95 ± 20 | 22 ± 5 |
| AS/G85R |  |  | 10 ± 14 | 110 |

Apo-SOD1 was incubated in 50 mM MOPS, 0.1 M NaCl, pH 7, plus 1 M guanidine as indicated. The time course of ThT fluorescence was fit to a sigmoidal equation and the amplitude and lag time determined as described in “Materials and Methods”. Dashes indicate that no rise in ThT fluorescence was observed.
